# Bioelectric and Morphological Response of Liquid-Covered Human Airway Epithelial Calu-3 Cell Monolayer to Periodic Deposition of Colloidal 3-Mercaptopropionic-Acid Coated CdSe-CdS/ZnS Core-Multishell Quantum Dots

**DOI:** 10.1371/journal.pone.0149915

**Published:** 2016-02-25

**Authors:** Aizat Turdalieva, Johan Solandt, Nestan Shambetova, Hao Xu, Hans Blom, Hjalmar Brismar, Marina Zelenina, Ying Fu

**Affiliations:** 1 Science for Life Laboratory, Department of Applied Physics, Royal Institute of Technology, SE-106 91 Stockholm, Sweden; 2 AstraZeneca R&D, SE-431 83 Mölndal, Sweden; 3 Advanced Light Microscopy facility, Science for Life Laboratory, Solna, Sweden; University of California, Merced, UNITED STATES

## Abstract

Lung epithelial cells are extensively exposed to nanoparticles present in the modern urban environment. Nanoparticles, including colloidal quantum dots (QDs), are also considered to be potentially useful carriers for the delivery of drugs into the body. It is therefore important to understand the ways of distribution and the effects of the various types of nanoparticles in the lung epithelium. We use a model system of liquid-covered human airway epithelial Calu-3 cell cultures to study the immediate and long-term effects of repeated deposition of colloidal 3-mercaptopropionic-acid coated CdSe-CdS/ZnS core-multishell QDs on the lung epithelial cell surface. By live confocal microscope imaging and by QD fluorescence measurements we show that the QD permeation through the mature epithelial monolayers is very limited. At the time of QD deposition, the transepithelial electrical resistance (TEER) of the epithelial monolayers transiently decreased, with the decrement being proportional to the QD dose. Repeated QD deposition, once every six days for two months, lead to accumulation of only small amounts of the QDs in the cell monolayer. However, it did not induce any noticeable changes in the long-term TEER and the molecular morphology of the cells. The colloidal 3-mercaptopropionic-acid coated CdSe-CdS/ZnS core-multishell QDs could therefore be potentially used for the delivery of drugs intended for the surface of the lung epithelia during limited treatment periods.

## Introduction

Mechanisms of cell toxicity of nanoparticles are being studied extensively [1–5]. In everyday life, nanoparticles may enter human body via skin, gastrointestinal tract and respiratory system, the respiratory system being considered to be the major gateway [1, 6, 7]. In primary human lung cells, nano-size CdSe-based quantum dots (QDs) were found to increase gene expression of proinflammatory cytokines, cause DNA damage and induce changes in genes associated with mitochondrial function [5]. However, little is known about the effects of the nanoparticles in human lung tissue regarding the fate of nanoparticles after they have landed in the lung, e.g., how rapidly they disperse on the surface of the lung, whether they can penetrate the glycocalyx layer covering the cells, and how they affect the integrity of the alveolar epithelial layer.

Human airway epithelial Calu-3 cell line is one of the few respiratory cell lines that form tight junctions *in vitro* which makes it a sensitive and efficient preclinical airway epithelial layer model for studying human respiratory processes [8–10], drug transport [11, 12], metabolic characteristics [13], mechanisms of lung injuries [14], and human rhinovirus infections [15]. Moreover, Calu-3 cell line provides an approximation of the *in vivo* situation of mechanical ventilation and oxygen toxicity better than many other *in vitro* models [14].

Tight junctions are critical for the formation and functioning of epithelial and endothelial barriers to regulate paracellular diffusion [16, 17]. Transepithelial electrical resistance (TEER) provides a good measure of the formation of the tight junctions and is often used as a marker of integrity and restrictiveness of the epithelial layer *in vitro*. Effect of polystyrene latex beads [18], metal-based nanoparticles [19], and carbon nanoparticles [20] on airway cells were studied using the TEER measurements.

In this work, we focus on the effect of periodic exposure of colloidal 3-mercaptopropionic-acid (3-MPA) coated CdSe-CdS/ZnS core-multishell QDs on the integrity of Calu-3 epithelial cell monolayers. We first validate the formation of the epithelial cell monolayers and the tight junctions. TEER measurements are then conducted both short-term and long-term after multiple deposition of the QDs. Immunocytochemistry is then used to find possible morphological changes in Calu-3 epithelial monolayer after repeated QD deposition.

## Materials and Methods

### Calu-3 cell culture

Human airway epithelial cells (Calu-3) purchased from ATCC (ATCC HTB-55TM [21]) were cultured using the protocol described previously [10] with slight modifications. Briefly, the cells were cultured in ATCC-formulated Eagle’s Minimum Essential Medium (EMEM, ATCC 30-2003) supplemented with 10% fetal bovine serum (FBS) and 100 units/mL of penicillin and 100 μg/mL of streptomycin in a humidified atmosphere with 5% CO_2_ at 37°C. The cells were seeded into cell culture Transwell-Clear inserts (6-well clusters, 24-mm inserts with polyester membrane, pore diameter 0.4 μm, Corning NY) at a density of 0.4 × 10^6^ cells/insert (0.085 × 10^6^ cells/cm^2^).

The medium in the inserts and in the compartments under the inserts (the “basolateral” compartments) was changed every 2 days at the beginning of the cell culture and every 3 days after the maturation of the cell monolayer. Cells were allowed to grow in the cell culture medium until their TEER had reached a plateau, indicating the formation of a tight epithelial cell monolayer, which was further validated by a three-dimensional confocal imaging using a Zeiss LSM 780 confocal microscope with a 63 × /1.4 oil objective. We then replaced the cell culture medium in the insert with simulated lung fluid (SLF) while the basolateral compartment remained filled with the cell culture medium to mimic the *in vivo* situation. The SLF was prepared according to formula SLF3 in [22], with Curosurf (porcine lung lipids and protein, Takeda Pharma, 80 mg/mL) added as a lung surfactant at a concentration of 0.0031%. The cells of passages 2–5 were used for the experiments.

### Immunocytochemistry

To visualize the cell structures, the cells were fixed using 4% paraformaldehyde (Acros Organics, Thermo Fisher Scientific), permeabilized in phosphate buffered saline (D-PBS, Thermo Scientific, VWR) containing 0.3% Triton X-100 (VWR), blocked using D-PBS with 5.0% goat serum (Life Technologies). The cells were subsequently incubated with one of the primary antibodies (see below) and a corresponding Alexa Fluor 488 conjugated secondary antibody. We used mouse monoclonal antibodies (BD Transduction Laboratories, Franklin Lakes, NJ) to recognize E-cadherin (protein associated with the tight and adherence junctions), rabbit polyclonal antibodies (Abcam, Cambridge, UK) to recognize occludin protein in the tight junctions, rabbit polyclonal anti-ezrin antibodies (Merck Millipore, Darmstadt, Germany) to stain microvilli, and Alexa Fluor 546 phalloidin (Molecular Probes, Thermo Fisher Scientific) to stain actin cytoskeleton. TO-PRO-3 Iodide and DAPI (Life Technologies, Thermo Fisher Scientific) were used to label nuclei.

Stained samples were studied using a Zeiss LSM 780 confocal microscope (Carl Zeiss, Jena, Germany) with a Plan Apochromat 63 × /1.4 oil DIC M27 objective and a 32-channel GaAsP spectral detector. Super-resolution structured illumination microscopy (SIM) was performed on a Zeiss ELYRA PS1 system using a 63 × /1.4 oil objective.

### Colloidal quantum dots

Water-dispersible 3-MPA coated CdSe-CdS/ZnS core-multishell QDs were prepared using common chemical synthesis method described in detail before [23]. Consisting of a CdSe core, a CdS shell of 2 monolayers, another shell of 1 monolayer Cd_0.5_Zn_0.5_S, and 1.5 monolayer ZnS, these QDs were coated with 3-MPA surface ligands and had a fluorescence peak at about 590 nm at room temperature. The diameter of the QDs without the 3-MPA surface ligands was about 5.7 nm. They were dispersed in deionized water (pH = 7.2) at a concentration of 12 μM.

Note that in the pilot studies of the work, we used QDs of various sizes ranging from 5.0 nm to 7.5 nm from different growth batches and did not observe any difference between their effects on Calu-3 cells.

### Bioelectric measurements, confocal microscopy of living cells, and QD detection

TEER of the epithelial cell monolayer was measured using an EVOM^2^ epithelial volt-ohm meter (World Precision Instruments, Sarasota, FL). The Transwell cluster was placed on a heating plate with a surface temperature of 37.0°C. The STX2 electrodes sterilized in 99.5% ethanol were gently placed into one of the three ports in the inserts so that one electrode had a contact with the medium above the cells, and the other with the medium in the basolateral compartment. For the long-term measurements, three measurements (one per port) were performed on each insert. For the short-term measurements, the TEER was recorded continuously for 20 min in the same port. The data are presented as TEER value (unit = kΩ⋅cm^2^), which is the TEER measurement (kΩ) × area of membrane (= *πr*^2^, where the radius of our inserts is *r* = 1.2 cm).

Distribution of QDs applied on the epithelial cell monolayers was studied by confocal and bright-field imaging of the inserts placed directly on a microscope slide using the confocal microscope using a 40 × /1.3 oil objective.

QDs’ penetration through the epithelial cell monolayers was studied by measuring QDs’ fluorescence from the media in the inserts and in the basolateral compartments using FluoroMax-3 spectrofluorometer (Horiba Jobin Yvon, Horiba Scientific, Kyoto, Japan). The excitation light wavelength was 400 nm, bandpass slits of the excitation and emission light were 5 nm, and the detection integration time was 0.3 s.

## Results

### TEER during the cell culture

As mentioned before, TEER is commonly used as a measure for the integrity of the epithelial cell layers *in vitro*. Cells are allowed to grow for 10–14 days until their TEER reaches a certain value (depending mostly on the cell line, passage and the Transwell type), indicating the formation of a tight epithelial cell layer. Reported values of the TEER vary also due to inter-laboratory differences in measurement procedure [11]. To characterize the TEER of our Calu-3 cell cultures, the TEER measurements were performed once a day starting on the next day after seeding of the cells on the Transwell inserts, which was referred to as day 1. The measurements were performed at the same time each day according to the following protocol (short-term monitoring):

- the Transwell cluster was taken from the incubator and placed on the heating plate
- the TEER was measured in one of the ports in an insert for 20 min
- the Transwell cluster was returned back to the incubator

**Fig 1:**
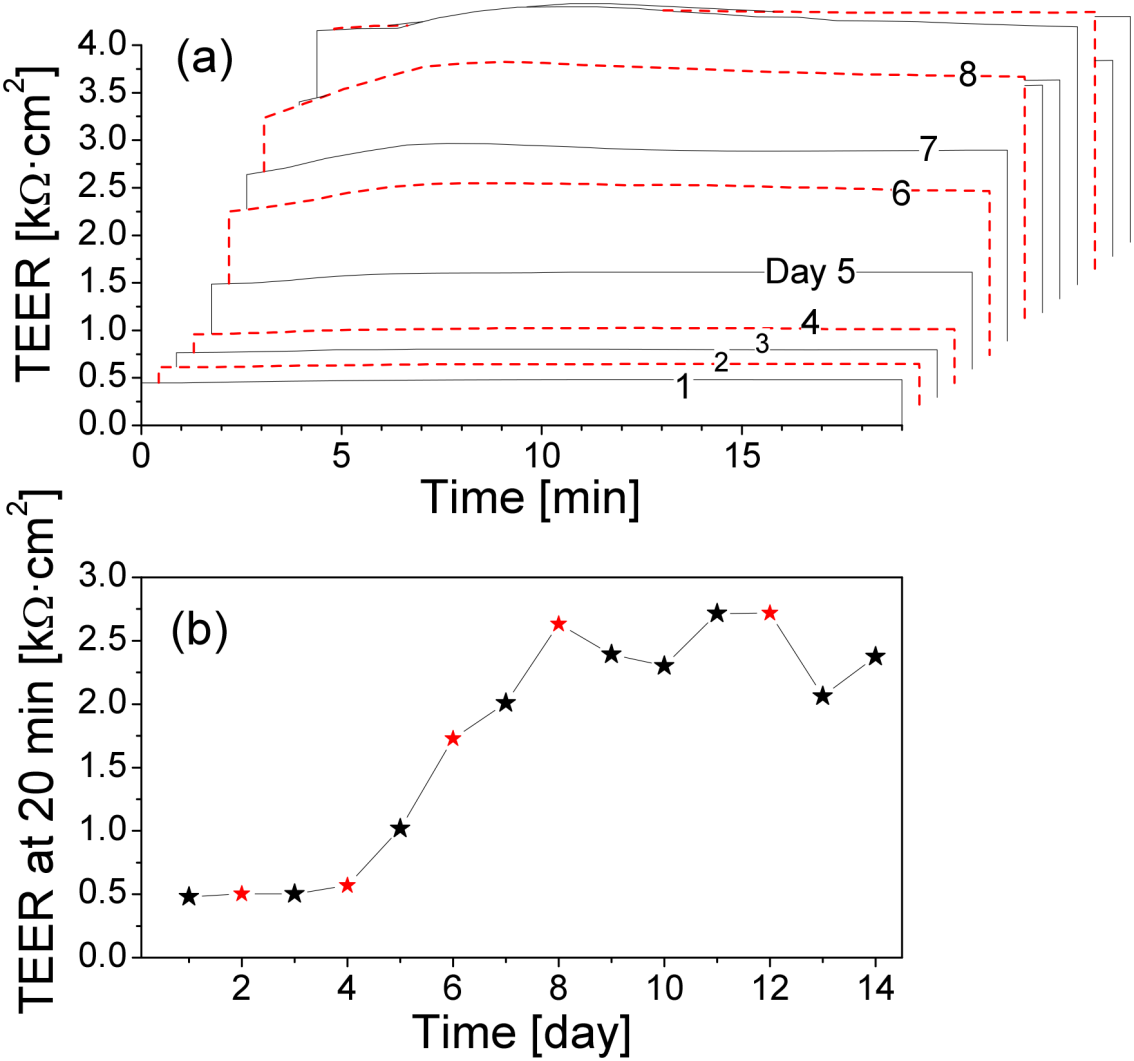
Evolution of the short-term TEER in Calu-3 cell monolayers during the monolayer maturation. (a) TEER measured for 20 min in the same port of the same insert on days 1–14 after the cell seeding. Red dashed lines indicate the TEER measured just before cell medium change. (b) TEER values at 20 min of the measurements in (a). Red symbols indicate the TEER measured just before the cell medium change. The mature cultures show a temporary decrease in the TEER after each medium change.

**Fig 2:**
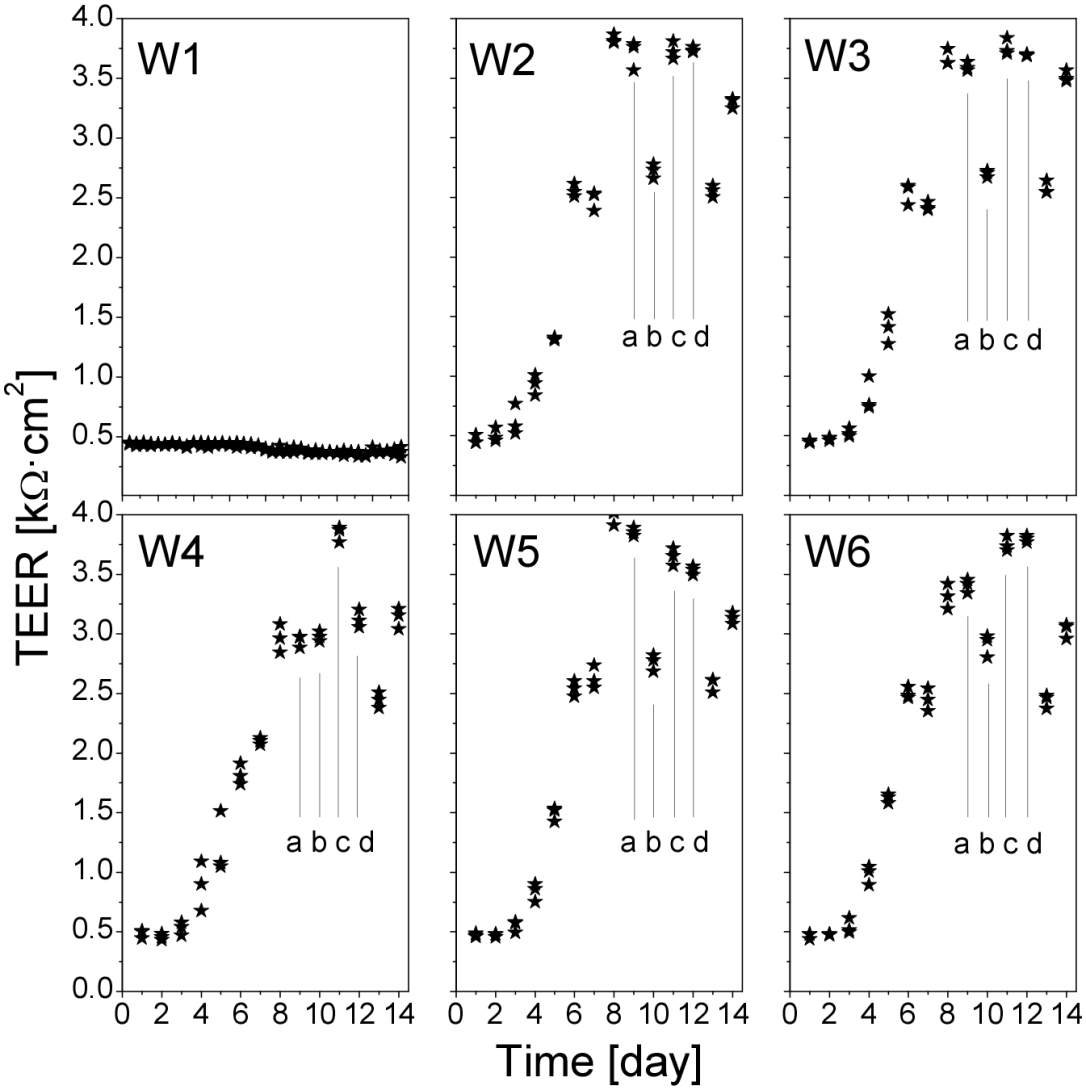
Long-term monitoring of TEER in Calu-3 cell monolayers. The measurements were performed in a Transwell cluster with six inserts (W1–W6, W1 was devoid of cells, acting as a reference). At each time point, the values are individual TEER measurements from the three ports of each insert. Vertical lines indicate the TEER (a) just before the medium change, (b) one day later, (c) two days later, (d) prior to the next medium change. The medium change affects the TEER drastically, with a recovery occurring two days later.

Typical results of the measurements are presented in Fig 1. The results showed that the TEER of the cell cultures started to increase noticeably from day 5 after seeding and reached its maximal value, about 3.6 kΩ⋅cm^2^, on day 10, while the TEER values of the blank inserts (devoid of any cells) were about 0.5 kΩ⋅cm^2^ (see W1 in Fig 2). Parallel confocal imaging studies (data not shown) revealed that on day 5 the confluence of the cells in the inserts was about 80%, and the confluent cell monolayer started to form on day 8. We observed that in the confluent cell monolayers the TEER values decreased dramatically on the first day after the cell medium change, which had to be taken into account later on when interpreting the TEER data of the cells during exposure to QDs.

Fig 1 also shows that the TEER varied during the time when the cells stayed outside the cell incubator. However, the TEER was relatively stable in the time window between 4 to 12 min of the measurements. We therefore used the following protocol for the long-term TEER monitoring:

- the Transwell cluster was taken from the incubator and placed on the heating plate for 5 min
- the TEER was measured once in each of the three ports in each of the six inserts in the Transwell cluster (18 TEER measurements within 2 min)
- the Transwell cluster was returned back to the incubator

Fig 2 shows the long-term TEER measurements in one of the Transwell clusters. The results demonstrated that upon the maturation of the cell monolayers the three TEER values in the three ports in each insert converged. The measurements have also confirmed our observation from the previous experiment that the decrease of the TEER after the medium change was characteristic only for the mature epithelial cell monolayers. In line with this, the cells in the insert W4, having lower and more dispersed TEER values on day 8, began to respond to the medium change later than in the other inserts in the same Transwell cluster.

**Fig 3:**
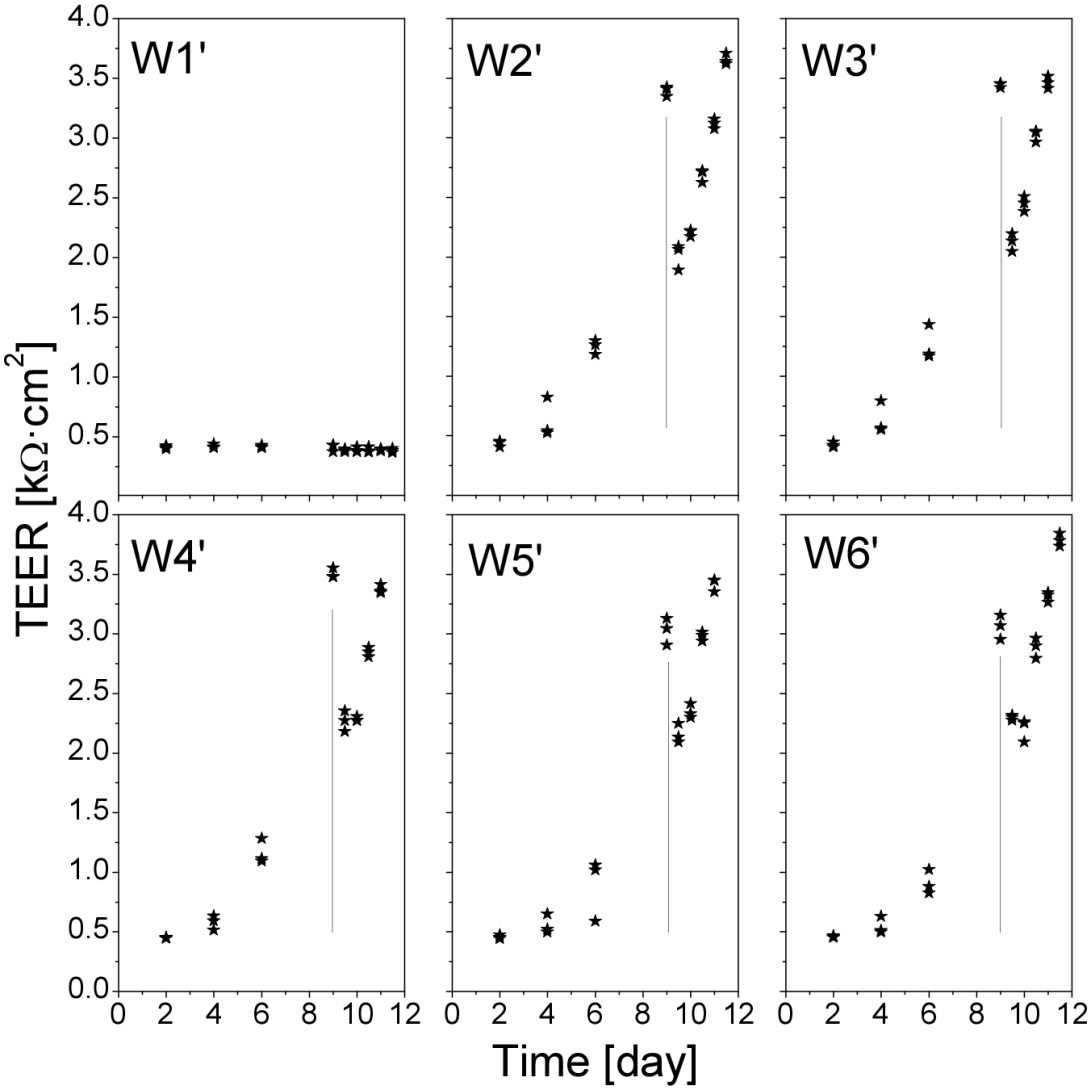
TEER of the Calu-3 cell monolayers measured at a 12-h interval. The measurements were performed in a Transwell cluster with six inserts (W1’–W6’, W1’ was devoid of cells). The values are individual measurements in the three ports of each insert. Vertical lines indicate the time just before the medium change. The TEER of the cell monolayers decreased already during the first 12 h after the medium change.

In order to more carefully assess the time of the recovery of the TEER decrease after the medium change, we performed a new experiment measuring the TEER every 12 hours. The results, presented in Fig 3, indicate that the TEER of the cell monolayers decreased already during the first 12 h after the medium change. The TEER values remained unchanged or changed very little after the following 12 h, then increased gradually over time.

### Morphological assessment of the epithelial cell monolayer maturity

**Fig 4:**
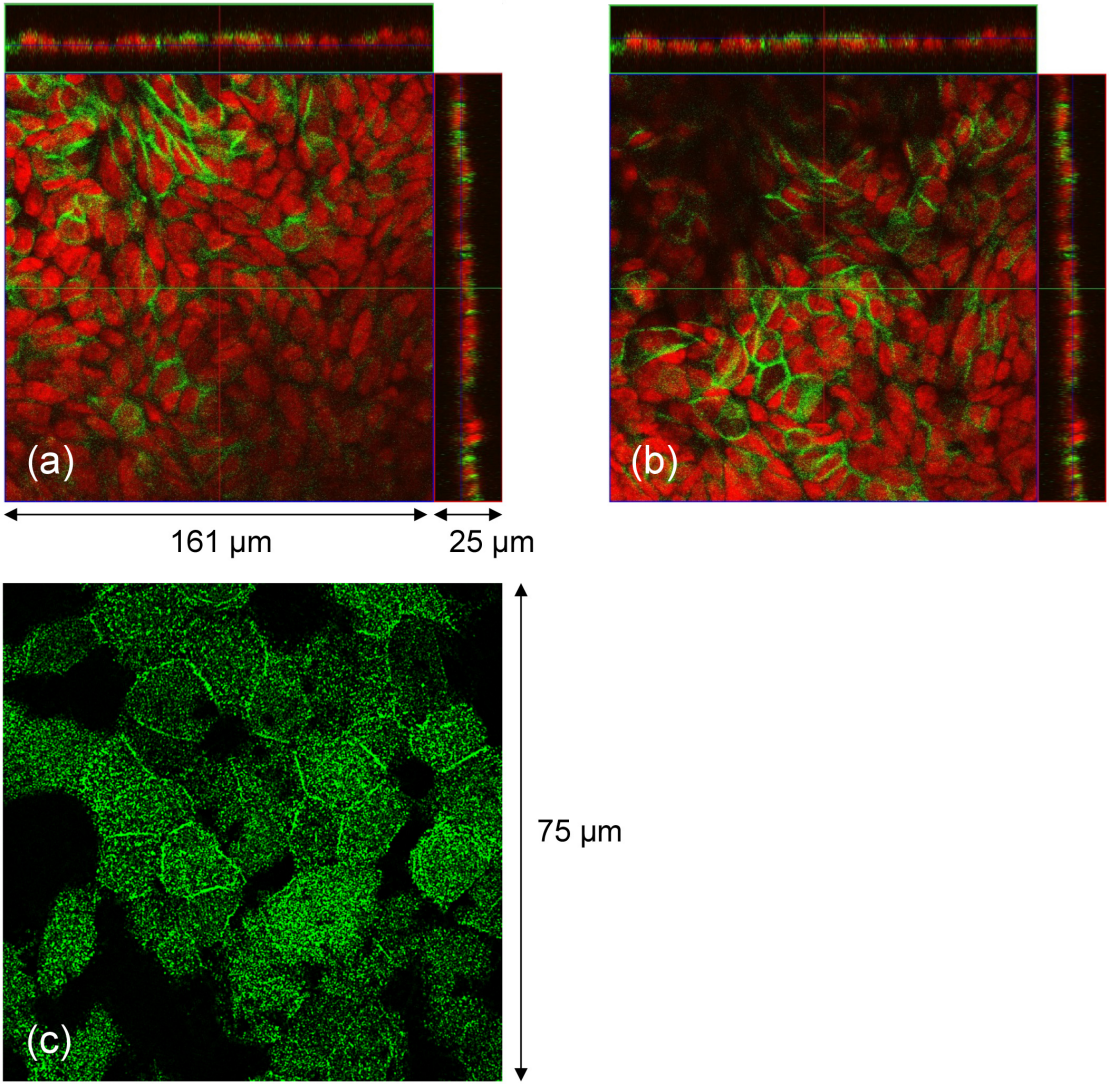
Molecular morphology of Calu-3 monolayers grown on the Transwell inserts (day 10 of the culture). (a,b) Confocal laser scanning microscopy imaging of E-cadherin (green) and cell nuclei stained with TO-PRO-3 (red). (a) and (b) show the same culture at two different *z* positions. Side panels show *z*-projections. (c) Structured illumination microscopy (SIM) image of ezrin protein distribution in microvilli. The distributions of both E-cadherin and ezrin indicated that the Calu-3 cell monolayers were well developed after 10 days in culture.

To assess the formation of the Calu-3 cell monolayers, we studied the molecular structures in the cells using immunocytochemistry and confocal microscopy. Fig 4(a) and 4(b) shows the staining for E-cadherin, the major proteins associated with the tight junctions and adherence junctions in epithelial cells [24]. The signals from the tight junctions were mostly located close to the apical surfaces of the monolayers. Fig 4(c) shows the staining for ezrin, protein present in microvilli at the apical surface of the epithelial cells [25]. The distribution of both E-cadherin and ezrin indicated that the Calu-3 cell monolayers were well developed in the Transwell inserts after 10 days in culture.

### Instantaneous and long-term effects of QD deposition on the TEER

We first studied the QD effects on the TEER of mature Calu-3 cell monolayers 14 days after the cell seeding using the following procedure:

- Step 1 (long-term TEER): In three Transwell clusters, A, B, and C (inserts A1–A6, B1–B6, C1–C6), we measured the TEER at 08.00, then changed the medium both in the inserts and in the basolateral compartments. The Transwell clusters were returned to the incubator. The TEER was then measured at 08.00 every day.
- Step 2 (short-term TEER): Five hours after the medium change, i.e., at 13.00, we measured the short-term TEER of insert A1 for 20 min. At 5 min, 50 μL of QD suspension (12 μM) was added to insert A1. The TEER was read once every 10 s between 5 and 8 min, and every 30 s during the rest of the 20-min period. The short-term TEER of insert B1 and C1 were measured in the same way with a deposition of 20 μL of QD suspension to insert B1, and of 50 μL of 3-MPA solution (10 mM) to insert C1 at 5 min.
- Step 3: One day after step 1, similar experiments were performed at 13.00 on insert A2 (50 μL QD suspension), B2 (20 μL QD suspension), and C2 (50 μL 3-MPA solution).
- Step 4: Two days after step 1, similar experiments were performed at 13.00 on insert A3, B3, and C3.
- Step 5: Three days after step 1, step 1–4 were repeated on inserts A4–A6, B4–B6, and C4–C6.
- Step 6: The cycle of steps 1–5 (six days long) was repeated. The whole experiment continued for two months.

There were several considerations that shaped the above experimental procedure.

1. The deposition of the 3-MPA solution was for the purpose of control. The concentration of 3-MPA was chosen based on the estimation that, according to the QD synthesis method, there were about 500 3-MPA ligands per each QD [26]. The concentration of 3-MPA in the 12 μM 3-MPA QDs was therefore 500 × 12 μM = 6 mM. In the experiments, the concentration of 3-MPA in its stock solution was 10 mM.
2. Adding 50 and 20 μL of 12 μM QD solution into the inserts corresponded to nominal QD concentrations of 387 and 158 nM, respectively. These were comparable with the QD doses used before [5] where QDs were added at concentrations of 20–160 μg/mL (each QD contains about 2000 II-VI atoms [27] and approximately 500 3-MPA ligands, resulting in a molecular weight of ca 150 kDa. 150 μg/mL corresponds to approximately 1 μM).
3. Most critically, the exposure to ambient air affects greatly the TEER of the Calu-3 cell monolayers. Due to this, each Transwell cluster was taken out from the incubator only twice a day, at 08.00 for 8 min (5 min on the heating plate to stabilize the TEER in ambient air, see Fig 1, then 2–3 min for the TEER measurement) and at 13.00 for 20 min.
4. The cycle of steps 1–5 was repeated to simulate the multiple exposure to the QDs as well as the recovery of Calu-3 cell monolayers from the periodic QD exposure.

**Fig 5:**
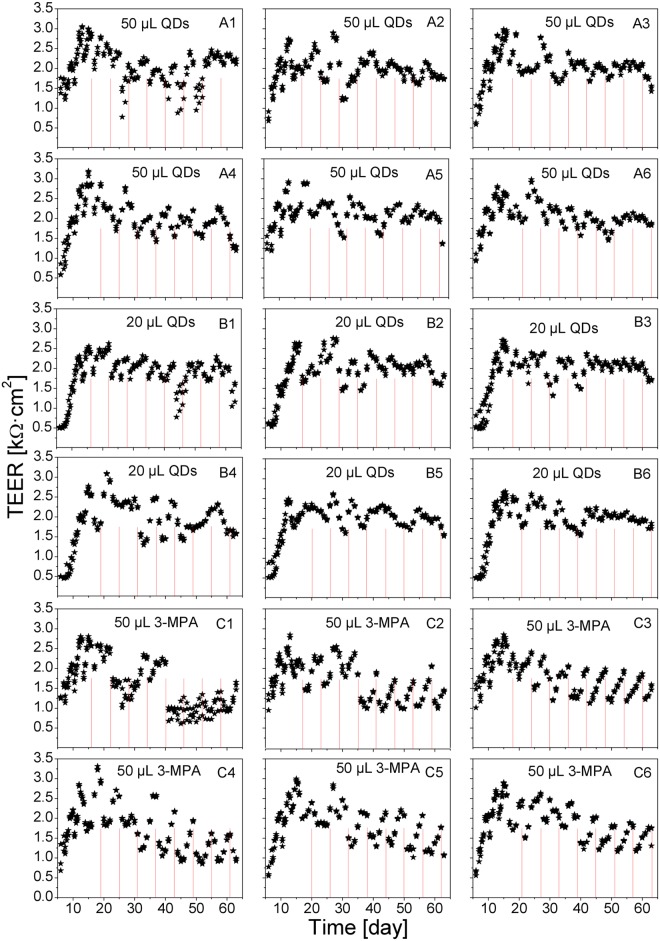
Long-term measurement of TEER of Calu-3 cell monolayers during multiple depositions of 3-MPA coated QDs. Red lines indicate the time points of the deposition of the QD suspension or 3-MPA ligands. At each time point, 50 μL of the QD suspension (12 μM) were added to the Transwell inserts A1–A6, 20 μL of the QD suspension to the inserts B1–B6, and 50 μL of the 3-MPA solution (10 mM) were added to the inserts C1–C6. Values are individual data points recorded from the three ports of each insert.

**Fig 6:**
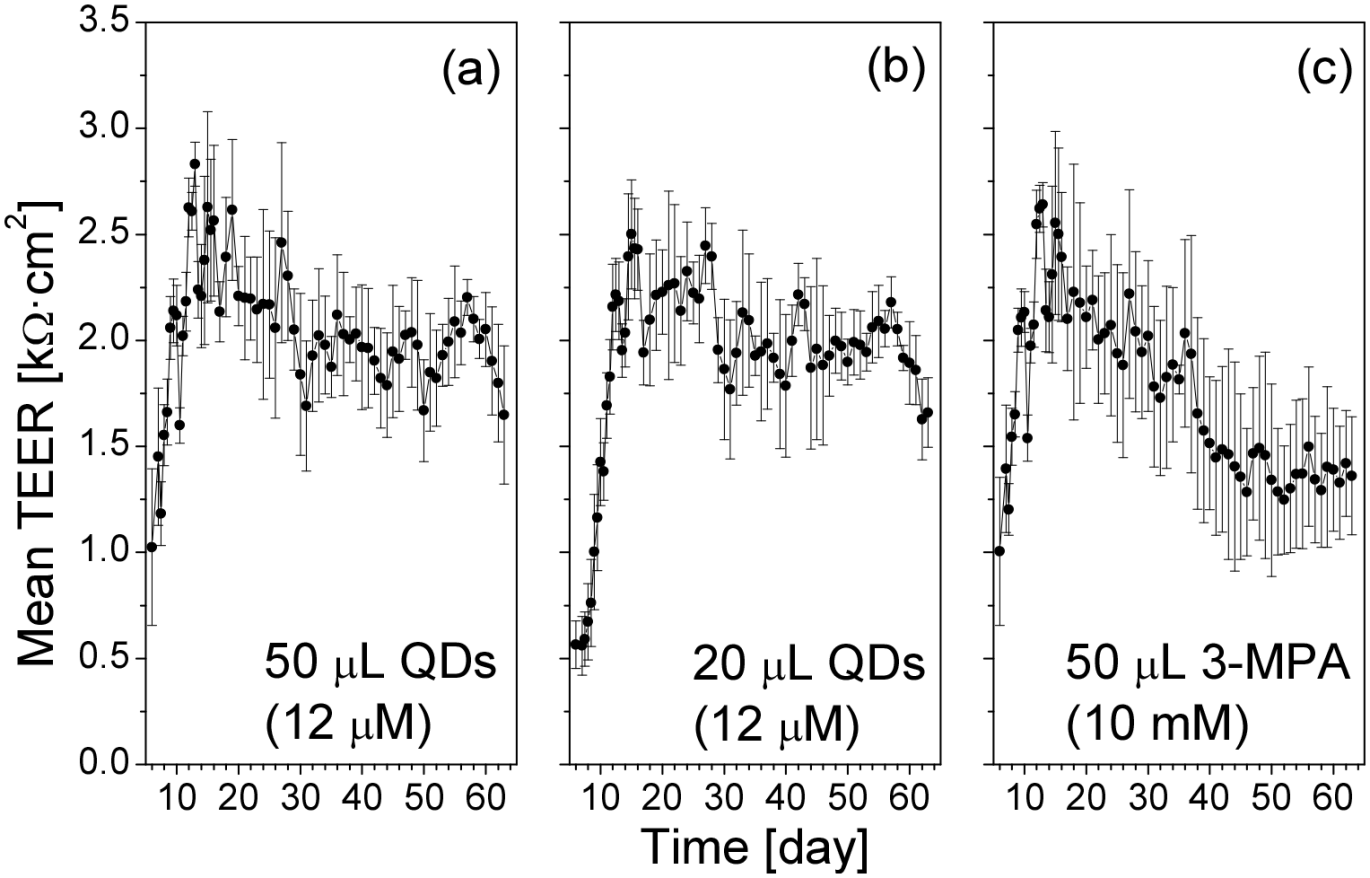
Averaged long-term TEER data from Fig 5. Values are means±SD from six Transwell inserts, with three data readings from each insert at each time point.

The long-term TEER results are presented in Fig 5 as individual measurements and in Fig 6 as averaged data. Individual inserts (such as A1 and C1) showed drastic TEER reduction, presumably due to a partial breakup of the monolayer at some time points, after which the long-term TEER recovered. We did not observe any clear difference in the general TEER dynamics depending on the time between the medium change (step 1) and the QD or 3-MPA deposition (the TEER time course compared between the inserts A1, A2, A3, and the group of inserts A4–A6; B1–B3 and B4–B6; C1–C3 and C4–C6, where the general drop in the TEER values in C1 on day 40 was clearly an artifact, most possibly a mechanical damage of the cell monolayer by the electrode).

All inserts demonstrated the characteristic TEER drops in response to each medium change. The time course of the long-term TEER recovery was not influenced by the applied QD amount. The deposition of 3-MPA led to a more pronounced variation in the long-term TEER values. One possible explanation for this is that the free 3-MPA was more biochemically active compared to the 3-MPA which was bound to QDs.

**Fig 7:**
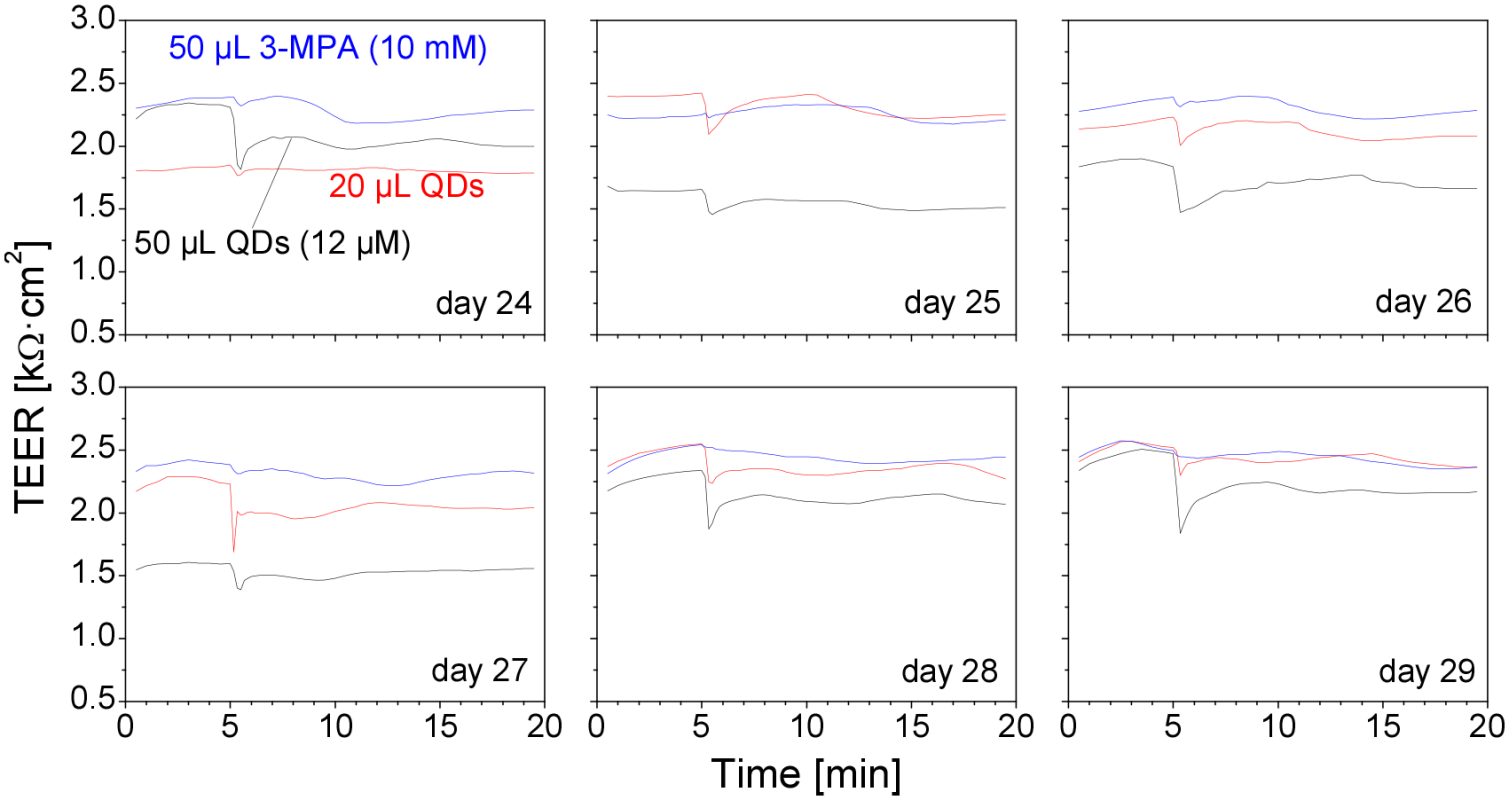
Effect of QD deposition on the short-term TEER of Calu-3 cell monolayers. Shown are representative TEER data recorded on six consecutive days. QDs (black lines, 50 μL of 12-μM QD suspension, red lines, 20 μL), and 3-MPA (blue lines, 50 μL of 10-mM 3-MPA solution) were added directly after the TEER reading at 5 min.

**Fig 8:**
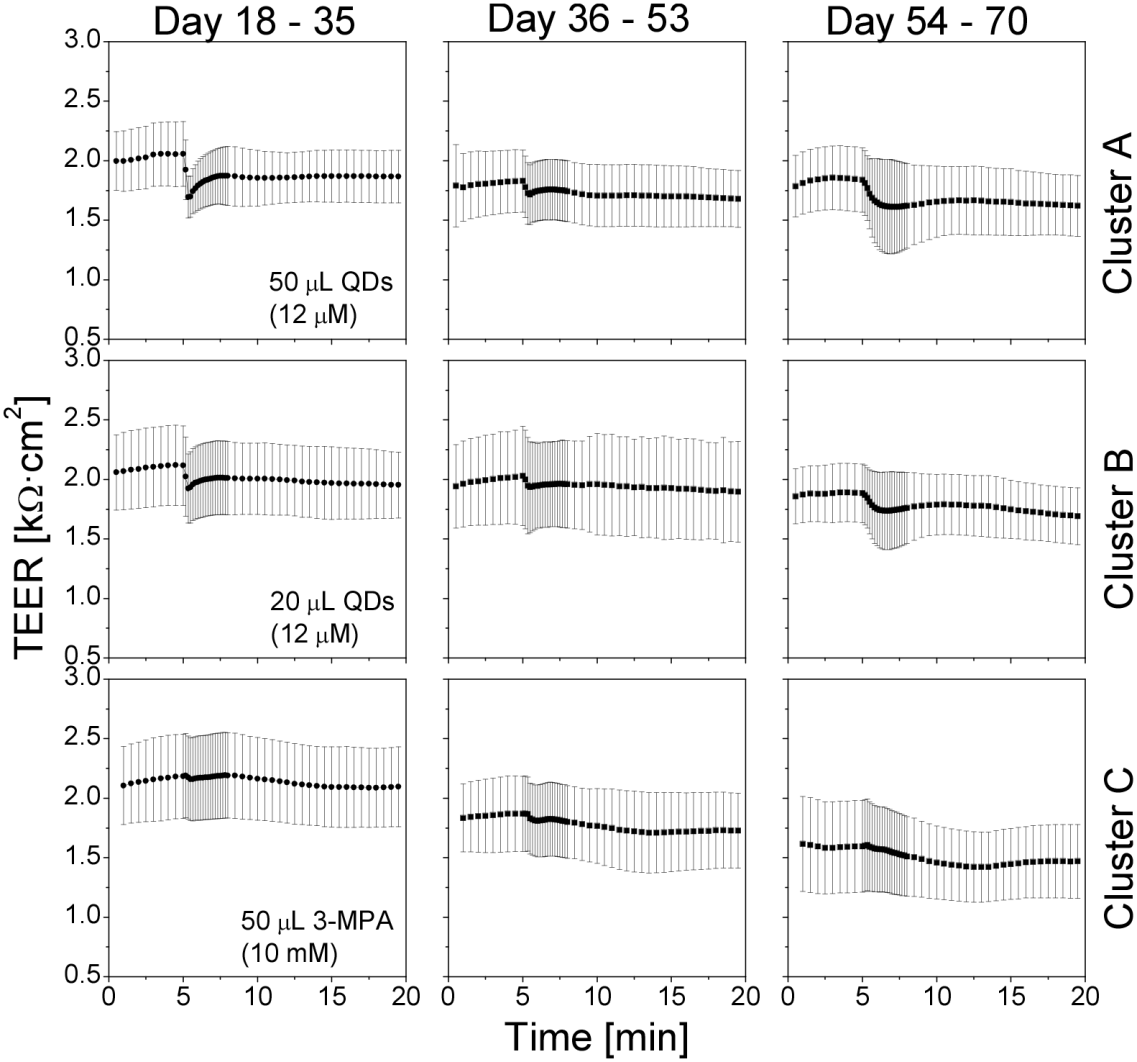
Average short-term TEER data in Transwell insert clusters A (upper row), B (middle row) and C (lower row). Values are means±SD from six inserts in each Transwell cluster. At each particular age of the cell cultures, there was no clear difference between the different QD doses with regard to the immediate effect on the TEER.

Representative short-term TEER data obtained in Transwell clusters A–C are shown in Fig 7. We observed a clear decrease in the TEER directly after the deposition of the QDs. Very small or no change was observed in response to the 3-MPA deposition. The averaged short-term TEER data are presented in Fig 8. The TEER values had a tendency to a decrease with the advancing age of the cell cultures, but at each particular age the amplitude of the TEER decrease induced by the QD deposition was more pronounced in the cells exposed to the higher QD dose. Deposition of the 3-MPA solution had the least immediate effect on the TEER values, but, similar to the effect on the long-term TEER dynamics (Figs 5 and 6), it had a clear influence on the average TEER level in the aging cultures.

**Fig 9:**
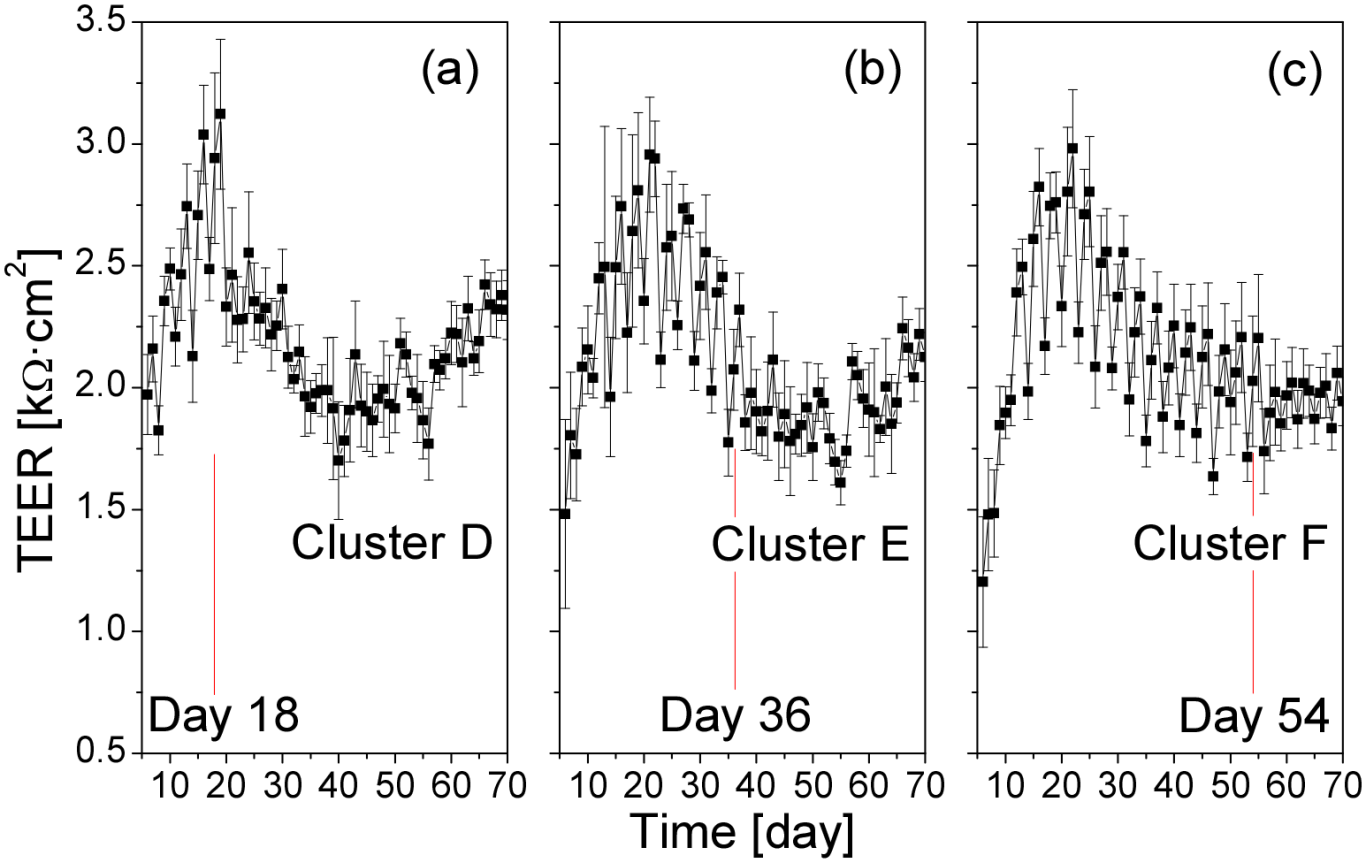
Effect of the cell culture age at the beginning of the QD treatment on the long-term TEER values. (a) Transwell insert cluster D was exposed to QDs starting from culture day 18, (b) cluster E was exposed to QDs starting from culture day 36, (c) cluster F was exposed to QDs starting from culture day 54. Values are means±SD from six inserts in each cluster. The red lines indicate the day of the start of the QD treatment. The cell culture age when the QD treatment started did not play any significant role in the long-term TEER changes.

Next, we examined the role of the cell culture age in the response to the QD exposure. We repeated the periodic QD exposure experiments in the following way: three Transwell clusters, D, E, and F, were seeded at the same day. At culture day 18, cluster D started to be treated by QDs (day 18: medium change at 08:00, adding 50 μL of QD suspension to insert D1 at 13:00; day 19: adding 50 μL of QD suspension to insert D2 at 13:00; day 20: adding 50 μL of QD suspension to insert D3 at 13:00; day 21: medium change at 08:00, adding 50 μL of QD suspension to insert D4 at 13:00; day 22: adding 50 μL of QD suspension to insert D5 at 13:00; day 23: adding 50 μL of QD suspension to insert D6 at 13:00; the process was repeated during the following days). On day 36, cluster E, and on day 54, cluster F, started to be treated with QDs in the same way. The averaged long-term TEER data from these experiments are presented in Fig 9. Apparently, the cell culture age when the QD treatment started did not play any significant role in the long-term TEER changes. The periodic modulation in the long-term TEER induced by the medium change became less pronounced in all three Transwell insert clusters after the start of the QD exposure, most clearly right after the beginning of the QD exposure. However, the average TEER values remained largely unaffected.

**Fig 10:**
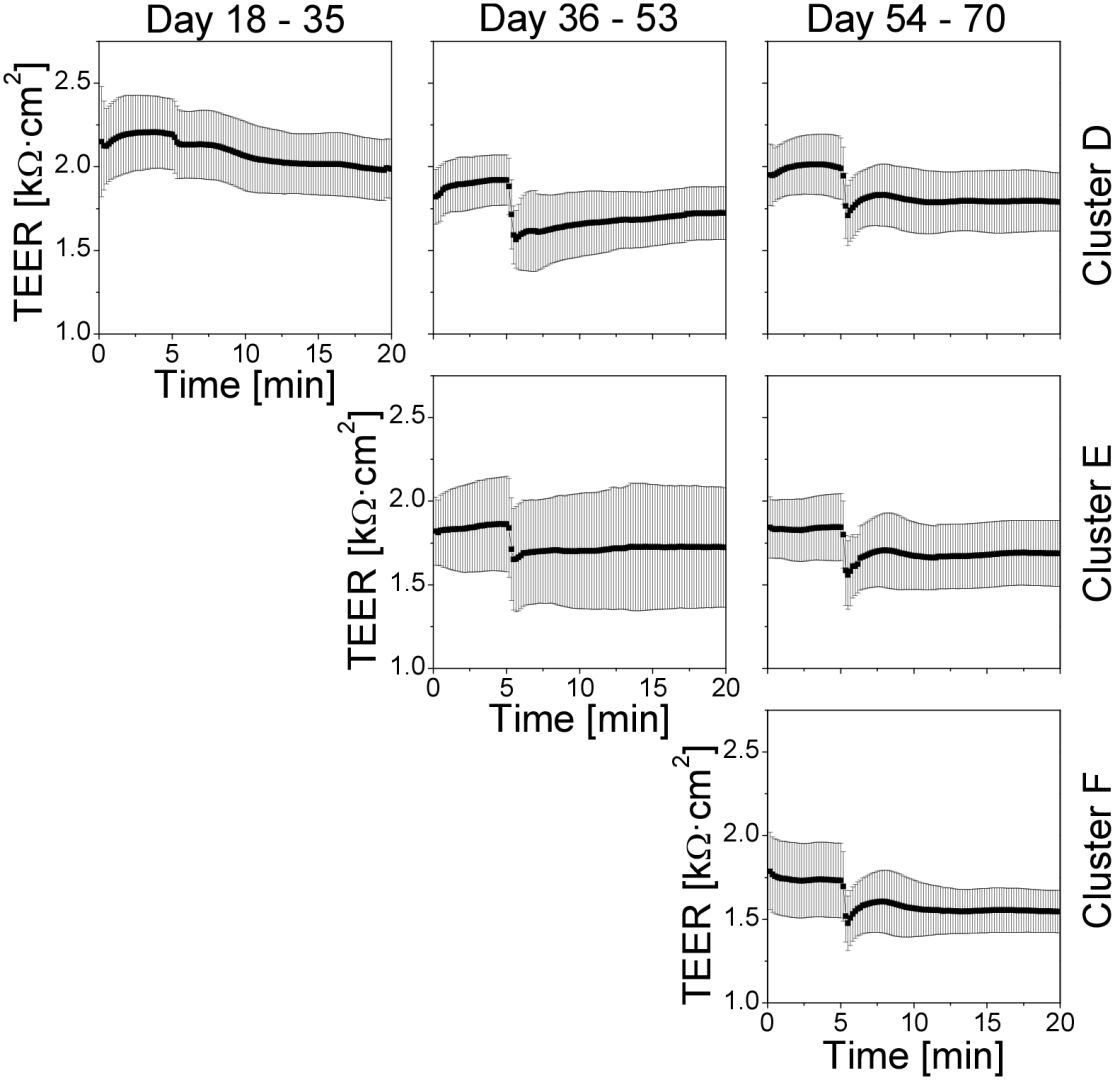
Effect of the cell culture age at the beginning of the QD treatment on the short-term TEER values. Upper row: insert cluster D was exposed to QDs starting from culture day 18; middle row: cluster E was exposed to QDs starting from culture day 36; lower row: cluster F was exposed to QDs starting from culture day 54. Values are means±SD from six inserts in each cluster (one TEER reading every 10 s). The TEER response to the QD deposition in 54–70 day old cultures was similar, independently of the previous QD treatment history.

The averaged short-term TEER from Transwell clusters D–F are presented in Fig 10. The QD response of the cells on the three Transwell clusters during day 54–70 did not display any clear dependence on the previous QD exposure history.

**Fig 11:**
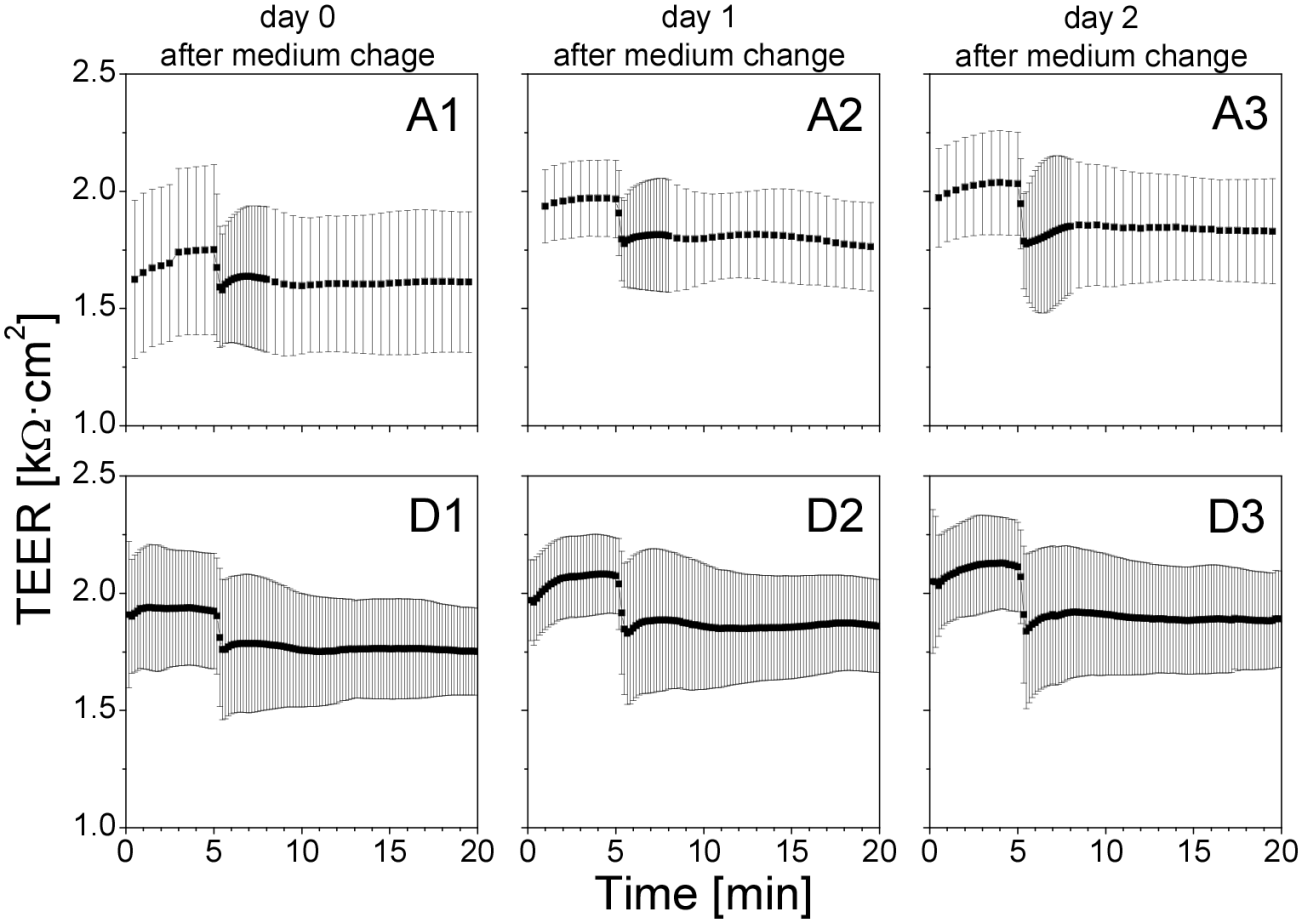
Short-term TEER of the cells exposed to QDs at a different time after the medium change. Inserts A1 and D1 were exposed to QDs a few hours after the medium change. Inserts A2 and D2 were exposed to QDs one day after the medium change. Inserts A3 and D3 were exposed to QDs two days after the medium change. The immediate effect of the QD deposition did not depend on the time that had passed since the medium change.

To examine the effect of the time between the medium change and the QD deposition on the short-term TEER, we have regrouped the data from Transwell insert clusters A and D, both treated with 50 μL of QD suspension starting from culture day 18, which are presented in Fig 11. The results indicate that the effect of the QD deposition on the short-term TEER values did not depend on the time that had passed after the “feeding” of the cells.

### QD distribution and effect on the cell morphology

**Fig 12:**
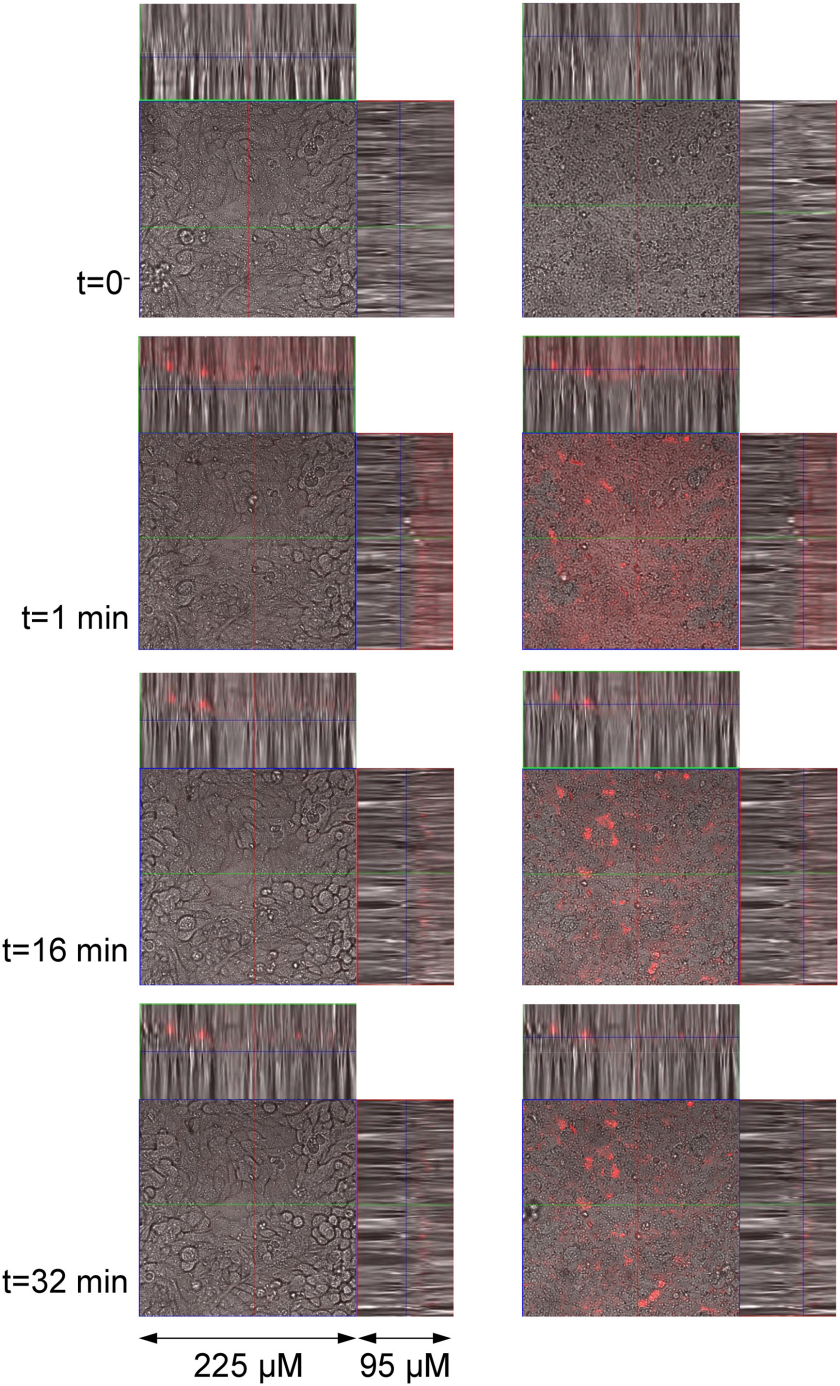
Confocal images of the living Calu-3 cells after the QD deposition. The QD fluorescence signal (red) is superimposed with the bright field image. Left and right panels show images taken at different depths of the cell monolayer: left panel images within the monolayer, right panel images slightly above the apical surface of the monolayer. The total observation time was 40 min. No significant penetration of the QDs into or through the cell monolayer was observed.

We monitored the distribution of QDs added to the Calu-3 cell monolayers under the confocal microscope in real time. A QD drop of 50 μL was added vertically above the center of the insert, with the objective positioned directly underneath the place of the QD deposition. A typical image sequence (Fig 12) shows the strong QD fluorescence signal above the Calu-3 monolayer at *t* = 1 min. During the observation time, the signal became weaker due to QD redistribution from the center to the periphery of the insert. After the recording had been finished, at *t* = 40 min, we examined the whole insert and found that the QDs were spread quite uniformly above the whole Calu-3 monolayer except a few randomly distributed QD clusters similar to the ones seen in the right-side image for *t* = 32 min. The obtained images suggest that within the 40 min of the observation the QDs did not penetrate into or through the cell monolayer. QDs sedimented on the apical surface of the monolayer, concentrated in some areas, due to gravity and/or active cilia movement.

**Fig 13:**
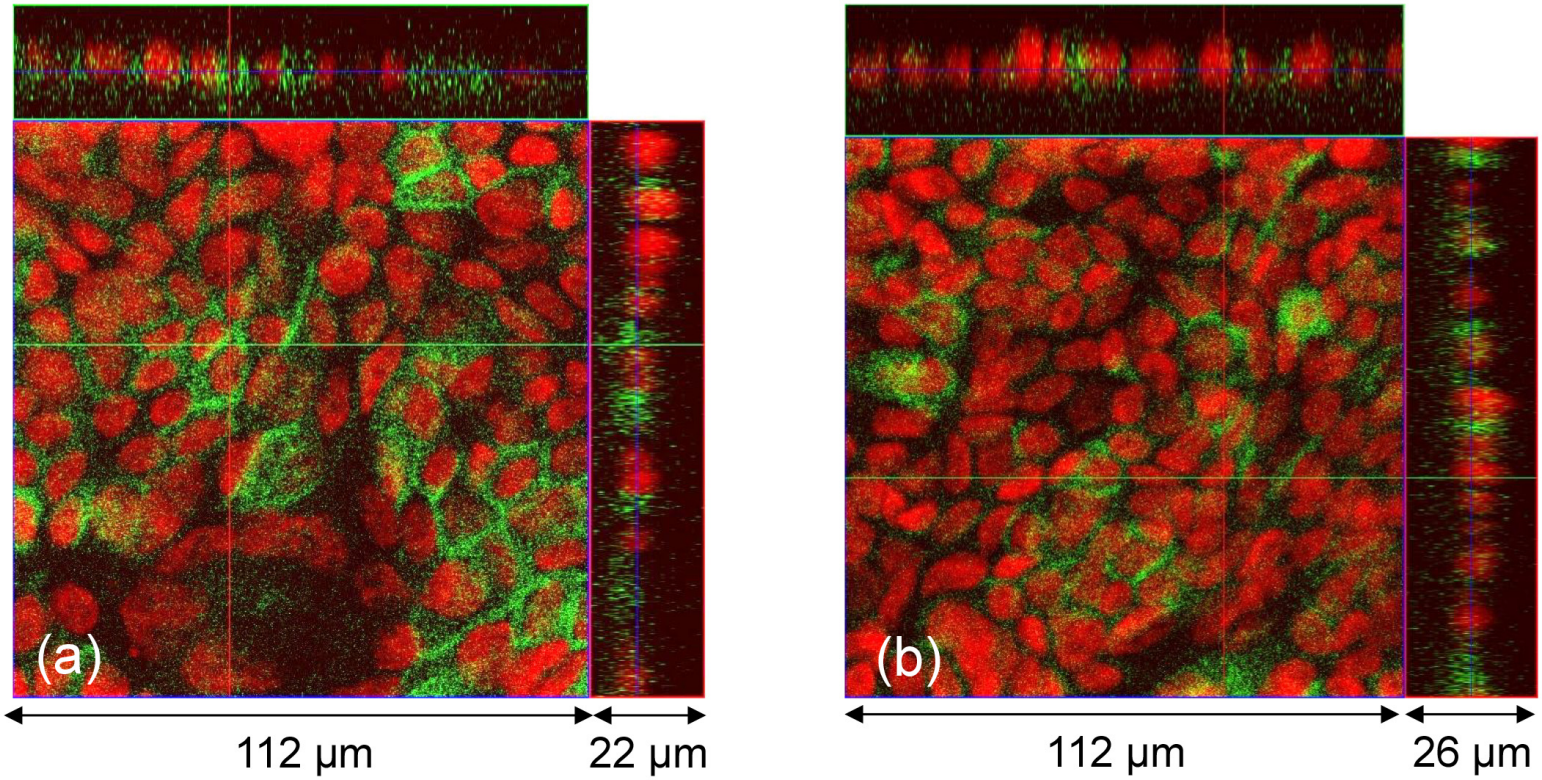
Molecular morphology of Calu-3 monolayers exposed to QDs (day 70 of the culture). (a) and (b) show the same culture at two different *z*-positions. Side panels show *z*-projections. Green: E-cadherin, red: nuclei. The multiple deposition of the QDs did not change the gross morphology of the cell monolayers (compare to Fig 4).

We stained the QD-exposed cells from the Transwell insert clusters A–F after 70 days of the long-term and short-term TEER measurements. Typical images of the stained cells are presented in Fig 13. By comparing with Fig 4 it was concluded that the morphological features of the epithelial cell monolayers remained largely unchanged by the multiple QD deposition.

**Fig 14:**
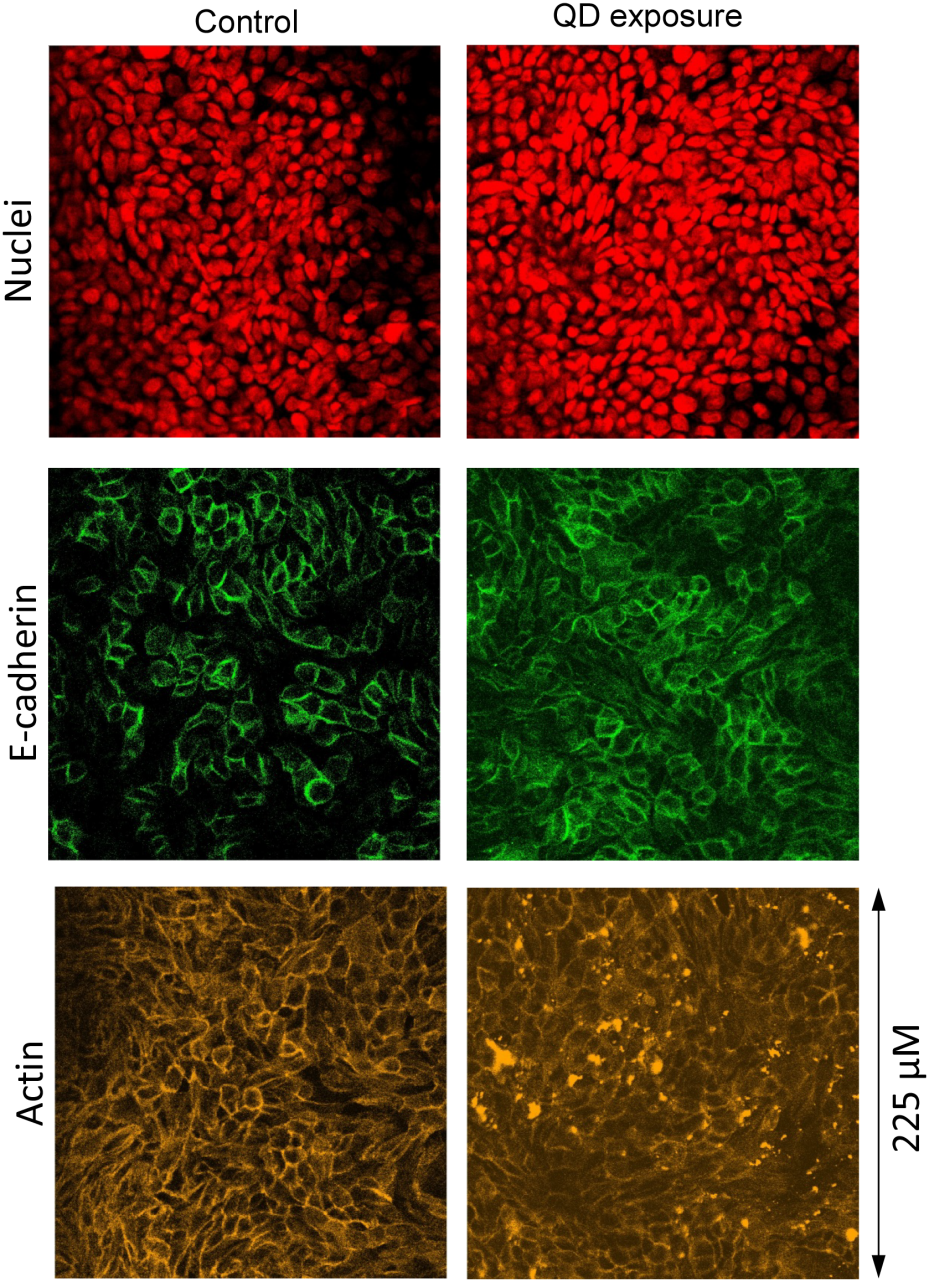
Confocal images of the control and QD-treated Calu-3 monolayers (day 70 of the culture). The cells were stained separately with TO-PRO-3 (nuclei), E-cadherin (tight and adherence junctions), and phalloidin (actin cytoskeleton). The multiple QD deposition did not cause any noticeable changes in the E-cadherin and actin distribution. The actin signal was somewhat disturbed by the strong fluorescence emitted by the QDs.

Another set of control and QD-exposed Calu-3 cell monolayers from this experiment were stained separately for nuclei, E-cadherin, and actin cytoskeleton (Fig 14). Again, the staining did not reveal any noticeable morphological changes in the QD-treated cells. The seemingly difference in the actin signal was due to the strong fluorescence emitted by the QDs that were present in the junction areas between the cells (seen after a single QD deposition, Fig 12, and within the cells after multiple QD treatment, see below).

**Fig 15:**
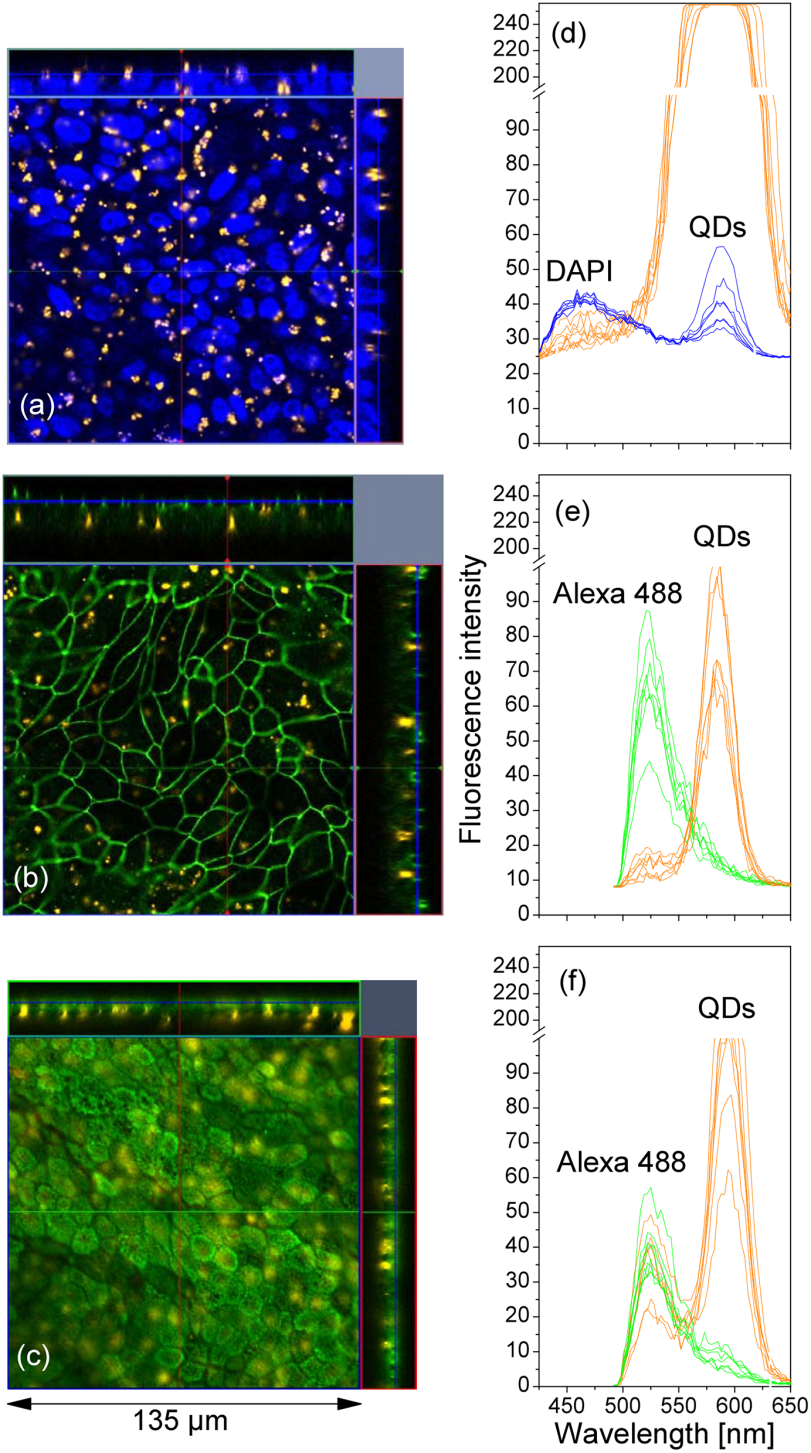
Distribution of QDs (orange) in Calu-3 monolayers after multiple depositions of QDs. (a) QD clusters did not co-localize with nuclei (DAPI, blue). (b) QD clusters did not co-localize with occludin (green). (c) QD clusters did not co-localize with microvilli (green). It can be concluded that the QDs aggregated in the perinuclear areas within the cells. (d-f) Optical spectra of a few fluorescence spots in the images of (a-c).

In order to study the distribution of QDs after repeated deposition on the cell monolayers, we examined the QD localization in cells stained for nuclei (DAPI), and for the markers of the tight junctions (occludin), and microvilli (ezrin). The representative results are shown in Fig 15(a)–15(c), where the fluorescence signals from QDs, nuclei (DAPI), occludin and ezrin (Alexa 488) were confirmed by their optical spectra in (d-f). The *z*-projection images clearly show that the QDs were localized within the cells, mostly near the nuclei.

**Fig 16:**
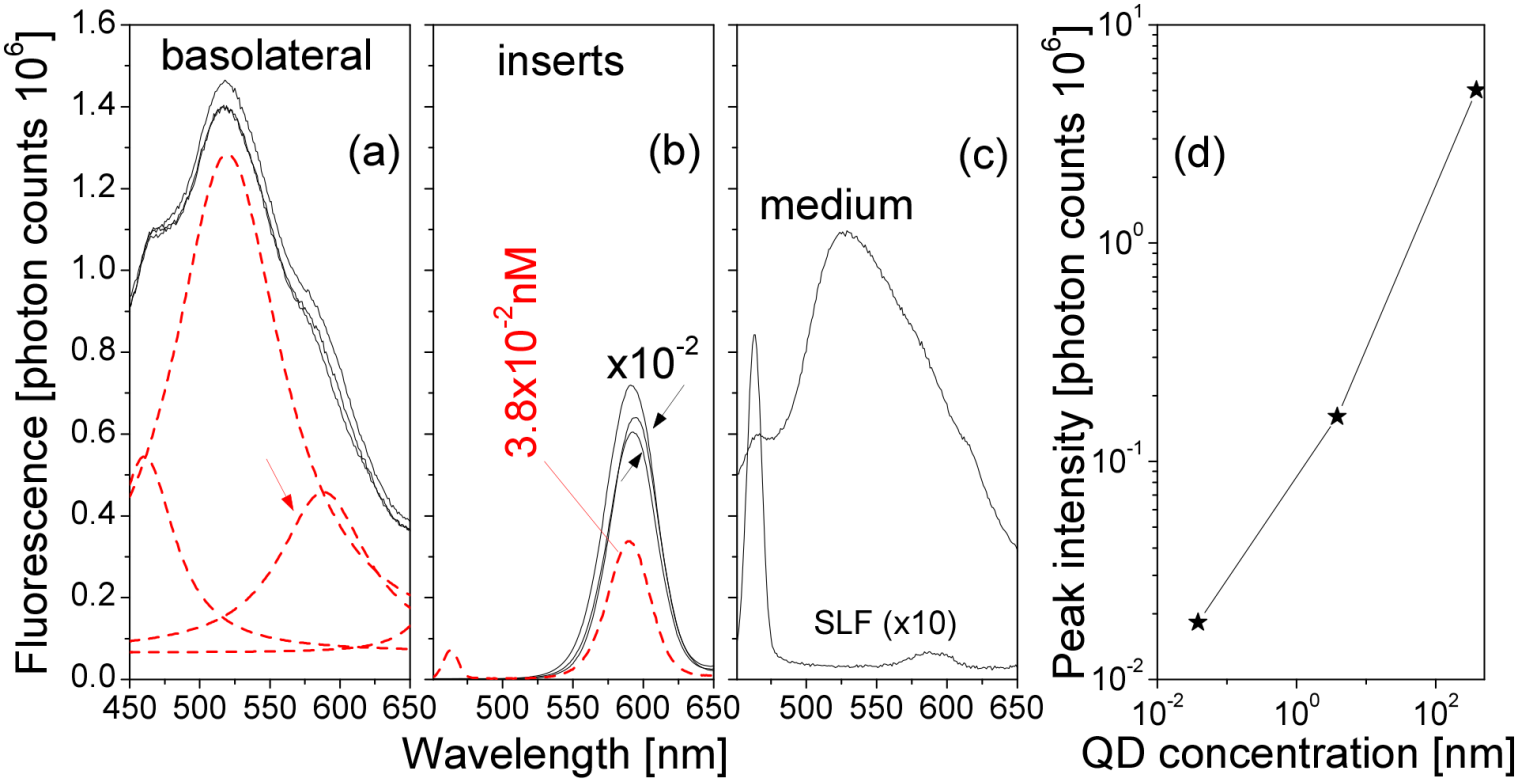
Fluorescence spectra of medium samples collected from the cell monolayers exposed to QDs for various time periods. (a) Medium collected from basolateral compartments. The black lines represent the spectra from three inserts exposed to QDs for one to three days. The red lines are fitted Lorentz peaks. (b) Medium collected from within the inserts (intensities scaled down by 10^2^). The red line denotes the fluorescence from diluted insert medium with a nominal QD concentration 3.8 × 10^−2^ nM. (c) Fluorescence spectra of pure cell culture medium and SLF. The SLF signal is scaled up 10-fold. (d) Peak intensity of the QD fluorescence versus nominal QD concentration.

The results presented in Fig 12 suggested that the QDs did not permeate significantly through the epithelial cell monolayer, at least within the observation time. In order to quantify the permeability of the QDs through the epithelial cell monolayers, we performed fluorescence spectroscopy measurements on medium samples collected during the medium change. In the morning of culture day 21, the medium in the Transwell cluster D was changed. Insert D4 was then exposed to QDs in the afternoon of day 21, insert D5 on day 22, and D6 on day 23. In the morning of day 24, during medium change, samples of the medium were collected from the inserts and from the basolateral compartments. The fluorescence spectra of the collected samples are presented in Fig 16. Despite the fact that insert D6 was exposed to QDs for less than one day, insert D5 was exposed for about two days, and insert D4 for almost three days, the spectra obtained from the medium samples in the three basolateral compartments were very similar. The fluorescence spectra of the samples were fitted with four Lorentz peaks (Fig 16(a)). The major peak and its left shoulder originated from the cell culture medium (compare with the medium peaks in Fig 16(c)). The right shoulder of the spectra in Fig 16(a) (590 nm) was due to the presence of trace amounts of QDs in the basolateral medium samples. A very weak 590-nm peak was also observed in the pure SLF solution (Fig 16(c)). However, this peak was too weak to be of any significance to the fitted peak in Fig 16(a). In the samples from the inserts, which contained SLF instead of the cell medium, the signal from QDs prevailed (Fig 16(b)). The peak at 460 nm characteristic for SLF became visible only when the QD suspension was diluted with pure SLF to the concentration of 3.8 × 10^−2^ nM.

In order to estimate the QD concentration in the medium in the basolateral compartments, we measured the peak intensity of the QD fluorescence in the original and diluted QD suspensions (Fig 16(d)). As mentioned before, we added 50 μL of the QD suspension (12 μM) to an insert containing 1.5 mL SLF solution, so that the nominal QD concentration in the insert was 387 nM. By comparing the intensity of the fitted peak in Fig 16(a) with the peaks in Fig 16(b) and the calibration curve in Fig 16(d), we estimated that the QD concentration in the basolateral compartments was about 2 × 10^−2^ nM, i.e., only 0.1% of the QDs permeated through the cell monolayers.

As a control, we added one QD drop of 50 μL (12 μM) to one blank insert and found similar strong QD fluorescence signals from both the insert and the basolateral compartment. The QDs with the size of about 8 nm in diameter including surface ligands freely penetrated the 0.4-μm pores in the polyester membrane of the insert.

Practically identical results were obtained from all the medium samples collected from the Transwell clusters A–F at different culture age (up to day 70, data not shown). It was therefore concluded that the QD permeability through the Calu-3 monolayer was very low.

### Discussion

This study was undertaken to understand the dynamic interaction of 3-MPA coated CdSe-CdS/ZnS core-multishell QDs with a lung alveolar epithelial cell layer. As a model, we used liquid-covered cultures of human airway epithelial Calu-3 cells.

In our hands, the Calu-3 cells grown on permeable polyester supports with 0.4-μm pores after 8–10 days in culture formed monolayers with well developed tight junctions and the TEER values of about 2.7–3.6 kΩ⋅cm^2^. The 14–70 days old cultures were used to explore the distribution and effects of single and multiple QD deposition.

The QDs were applied on the cells by adding a drop of the QD suspension to the liquid in the apical compartment of the Transwell inserts. In response to the QD application, we observed an immediate transient TEER decrease. This decrease was dose-dependent, was present in Calu-3 monolayers of different age, both in cells being exposed to the QDs for the first time or those that were already exposed to the QDs repeatedly. It could be suggested that the decrease in the TEER values was a result of a temporary increase in the transepithelial conductance due to ciliary-mediated mechanosensation. Calu-3 cells were shown to form primary or multiple cilia at the apical side of the monolayers [28]. It is well known that the primary cilium of epithelial cells, being a liquid flow sensor in many cells, can transduce an externally applied mechanical signal to intracellular signaling pathways (reviewed in [29]). One of the effects of the mechanical stimulation of cilia is an increase of the intracellular Ca^2+^ concentration [29, 30]. In turn, the agents that increase the intracellular Ca^2+^ concentration were shown to induce an increase in transepithelial conductance and transepithelial currents in Calu-3 monolayers [31, 32]. The underlying mechanism is an increase of potassium outward currents via Ca^2+^-activated K^+^ channels at the basolateral membrane of the cells, which activates chloride secretion at the apical plasma membrane via cystic fibrosis transmembrane conductance regulator (CFTR) Cl^−^ channels. This mechanism was also found to be behind the increase of the transepithelial current increase induced by negatively charged polystyrene nanoparticles [33]. The suggestion that the deposited QDs, by mechanical stimulation of the cell cilia, induced a Ca^2+^-dependent increase in the transepithelial conductance was supported by our observation that the effect was more pronounced with the increase of the concentration of the deposited QDs and by the fact that even the application of 3-MPA solution lead to a small transient TEER decrease by disturbing the solution above the cells.

The ciliary-mediated mechanosensation might also contribute to the dramatic decrease in the TEER values in the mature Calu-3 cell monolayers after each change of the cell medium. This phenomenon has not been reported before. A clear temporary (for 6–12 h) decrease in the resistance across the Calu-3 cell cultures was shown in a study of the effects of metal oxide nanoparticles [34], where the authors added the nanoparticles to the apical compartment of the inserts by a complete replacement of the cell medium by a test nanoparticle suspension. However, no comments were made concerning the initial drop of the resistance in those experiments. The baseline changes in the TEER due to changes/addition of solutions to the apical surface of the Calu-3 cultures might also be suspected in other studies. For example, an increase in TEER was observed in a study of the effect of polystyrene particles on Calu-3 cultures, where the TEER was measured after replacing the culture medium with a medium with the particles and compared to the TEER measured 3–5 days later [18]. A high variability in the bioelectric properties in the Calu-3 cell layers older than 20 days were also reported by Mathia et al. [12]. Obviously periodic changes of the TEER in 8–20 day old cultures in that study, very similar to the ones shown in Fig 1(b), might as well have been due to the medium changes. However, the time points of the medium changes were not indicated by the authors.

Besides the purely mechanical disturbances of the cultures during the medium changes, renewal of the solutions around the cells might disturb the cell microenvironment by washing out the biologically active substances that the cells produce, disrupting the ion gradients that they create etc. For example, an electrical potential difference of 11–16 mV has been reported to be present across mature Calu-3 monolayers [12]. Washout of the external solutions would abolish this potential difference, affecting various cell functions. In any case, the phenomenon of the severe TEER drops after the medium change should always be considered when planning experiments in cells grown on the Transwell inserts.

In our experiments we have found that the QDs deposited on the apical surface of the Calu-3 cell cultures were quickly distributed to areas of cell-to-cell junctions. Some signals were also observed in clusters above some cells of the monolayers. Calu-3 cell cultures are known to be comprised of a mixed phenotype of ciliated and secretory cells (reviewed in [12]). The ciliated cells in lung epithelia are known to be able to propel forward the liquid that covers the cells [35] and thereby to “brush out” the dust particles and bacteria that precipitate on the surface from the inhaled air. Similar action could be expected from the lung epithelial cells in culture. This would, in a closed space of a Transwell insert, lead to the accumulation of the QDs in cilia/microvilli-free areas.

The penetration of QDs through the Calu-3 cell monolayers in our cultures was found to be very low. No studies on the transport of QDs through the Calu-3 cell cultures have been performed before. However, our results are in a good agreement with studies using other kinds of particles on this cell line. The translocation of the metal oxide nanoparticles through the Calu-3 cell cultures was found to be very low, less than 0.01% of the applied dose after 24 h [34]. No translocation of carboxyl- or amine-modified polystyrene particles could be detected through Calu-3 cells cultured in the same conditions as in our study [18].

The exact route of those few QDs that were able to cross the Calu-3 cell monolayers in our study remains to be established. Based on the electron microscopy observations of the metal oxide nanoparticles within the Calu-3 cells, it was suggested that the small amounts of the particles that were translocated through the Calu-3 cell cultures were delivered there by a transcellular pathway [34]. However, no evidence was provided that the internalized particles were released by the cells on the basolateral side of the cells. No QD uptake studies in Calu-3 cells were reported so far, but the ability of cells to internalize QDs has been demonstrated in several other cell types. The uptake is usually significantly promoted by conjugation of the QDs with specific peptides that target them to the cell surface [36]. The internalized QDs often remain in the endosomal/lysosomal compartments of the cells. However, it is possible, by an advanced surface functionalization, to promote escape of the QDs from the endosomal compartments to other compartments within the cells [37], and further for exocytosis from the cells [38–40].

In experiments with the multiple deposition of QDs on the Calu-3 cultures we have found an accumulation of QDs in large clusters within the cells. Obviously, only a small fraction of the QDs from the deposited suspension were taken up by the cells (with the rest of them being washed out during the regular medium changes before adding a new dose). However, without any specific targeting molecules conjugated to our QDs, the cells were unable to effectively expel these QDs by exocytosis. This underlines significance of a proper biologically relevant labelling of QDs meant for delivery into or through the cells. Our QDs could instead be used for a delivery of a cargo to the surface of the cells. Our results suggest that the QDs could be used for this purpose repeatedly, since the accumulation of the QDs after the multiple deposition did not significantly influence the integrity of the Calu-3 cell cultures.

The estimated 500 3-MPA ligands per QD mentioned before was obtained according to the QD synthesis method [26]. There is unfortunately no effective experimental means currently to control this number. Variations of the coating composition, including labeling the QDs with specific targeting molecules, should be carefully studied when the QD surface coating structure can be properly measured. In this work we focused on the effects of the coated QDs as such.

A common concern of using QDs for biological use is the possible deterioration of the outer shell or potential leaking of toxic metal ions. We did spectral measurements on QDs in randomly chosen medium samples collected during medium changes as well as stained samples stored at +4°C for 6 months and found no perceptibe changes. All the chemical processes have a higher speed at +37°C compared to +4°C, but the absence of any perceptible changes in the QD spectra in the samples stored for 6 months at +4°C suggests that there would not be any significant deterioration of the QDs in the cell cultures exposed to the QDs for six weeks +37°C.

We might expect effects of Ag ions from the Ag/AgCl electrodes of the TEER measurement. However, there were several observations that suggest that the overall effect of the Ag ions on the TEER was hardly noticeable. For example, the TEER of the blank inserts (W1 in Fig 2 and W1’ in Fig 3) did not display perceptible changes, neither after the medium change nor after several measurements. Also, in the short-term TEER measurements where the electrode was submerged in the cell media for 20 min (Figs 7, 8, 10 and 11), we did not observe any consistent changes in the TEER values, except for the moment of QD deposition.
